# Identification of Prominent Genes between 3D Glioblastoma Models and Clinical Samples via GEO/TCGA/CGGA Data Analysis

**DOI:** 10.3390/biology12050648

**Published:** 2023-04-25

**Authors:** Brandon Wee Siang Phon, Saatheeyavaane Bhuvanendran, Qasim Ayub, Ammu Kutty Radhakrishnan, Muhamad Noor Alfarizal Kamarudin

**Affiliations:** 1 Jeffrey Cheah School of Medicine and Health Sciences, Monash University Malaysia, Jalan Lagoon Selatan, Bandar Sunway 47500, Malaysia; 2 School of Science, Monash University Malaysia, Bandar Sunway 47500, Malaysia; 3 Monash University Malaysia Genomics Facility, Monash University, Bandar Sunway 47500, Malaysia; 4 Tropical Medicine and Biology Multidisciplinary Platform, Monash University Malaysia, Bandar Sunway 47500, Malaysia

**Keywords:** 3D culture, Glioblastoma, differential expression, EMT, TCGA, CGGA, therapy resistance

## Abstract

**Simple Summary:**

Glioblastoma multiforme (GBM) is a deadly brain tumour with little progression in the way of improved quality of life in patients. The possible root cause for this dilemma stems from the inefficiency of traditional two-dimensional (2D) cell-based models used in evaluating potential anticancer agents. In this paper, we investigated the proximity with which three-dimensional (3D) GBM models encapsulate key aspects of clinical GBM samples that have been made available in many genetic data banks. The analysis identified a number of key genes highly expressed in both 3D GBM cell-based models and GBM clinical samples that may play a role in GBM therapy resistance. In conclusion, the findings suggest that 3D GBM cell-based models serve as reliable models for clinical GBM samples.

**Abstract:**

A paradigm shift in preclinical evaluations of new anticancer GBM drugs should occur in favour of 3D cultures. This study leveraged the vast genomic data banks to investigate the suitability of 3D cultures as cell-based models for GBM. We hypothesised that correlating genes that are highly upregulated in 3D GBM models will have an impact in GBM patients, which will support 3D cultures as more reliable preclinical models for GBM. Using clinical samples of brain tissue from healthy individuals and GBM patients from The Cancer Genome Atlas (TCGA), Gene Expression Omnibus (GEO), Chinese Glioma Genome Atlas (CGGA), and Genotype-Tissue Expression (GTEx) databases, several genes related to pathways such as epithelial-to-mesenchymal transition (EMT)-related genes (*CD44*, *TWIST1*, *SNAI1*, *CDH2*, *FN1*, *VIM*), angiogenesis/migration-related genes (*MMP1*, *MMP2*, *MMP9*, *VEGFA*), hypoxia-related genes (*HIF1A*, *PLAT*), stemness-related genes (*SOX2*, *PROM1*, *NES*, *FOS*), and genes involved in the Wnt signalling pathway (*DKK1*, *FZD7*) were found to be upregulated in brain samples from GBM patients, and the expression of these genes were also enhanced in 3D GBM cells. Additionally, EMT-related genes were upregulated in GBM archetypes (wild-type *IDH1^R132^* ) that historically have poorer treatment responses, with said genes being significant predictors of poorer survival in the TCGA cohort. These findings reinforced the hypothesis that 3D GBM cultures can be used as reliable models to study increased epithelial-to-mesenchymal transitions in clinical GBM samples.

## 1. Introduction

Glioblastoma multiforme (GBM), a grade 4 adult-type diffuse glioma, is the most common malignant and clinically aggressive brain tumour, and it is notorious for its unrelenting aggressiveness. The standard therapy against GBM, known as the Stupp protocol, involves surgical resection ranging from a minimally invasive biopsy to a craniotomy for maximal safe resection followed by radiotherapy and concurrent administration of temozolomide (TMZ) [1]. Reliable as it is, the Stupp protocol has only produced meagre improvements in GBM median survival, i.e., extending the median survival by an additional two months from the initial 12.1 months [1]. Although it has been two decades since the establishment of the Stupp protocol, the median survival of GBM patients has scarcely improved. The latest Central Brain Tumor Registry of the United States (CBTRUS) report documented the lowest overall survival for GBM patients at a mere eight months, a number lower than most brain neoplasms’ overall survival by at least half [2]. The high failure rate of anticancer agents used is one of the stumbling blocks in GBM therapy, as only 10% of drugs in preclinical evaluations make it to the clinic [3].

Across the globe, two-dimensional (2D) cultures remain the in vitro model of choice for preclinical evaluations. Recent advances in the cell culture approach, such as 3D cultures, have placed the widespread use of 2D cultures under intense scrutiny [4,5]. Despite its ease of use, the unnatural geometric and mechanical constraints imposed in a 2D environment come with several drawbacks that result in a lower resemblance of actual GBM phenotypes. These include the absence of tumour cell interactions, low similarity to actual tumour phenotypes, and unrestricted availability of oxygen, nutrients, and metabolites, which do not accurately replicate actual tumour microenvironments. Furthermore, the tumour microenvironment has been postulated to directly influence the genomic landscape of GBM tumours, which underlie the aggressive phenotype that confers higher resistance to therapy [5,6,7]. Additionally, immune cells, stromal cells, endothelial cells, mesenchymal cells, and the extracellular matrix in the cellular milieu also contribute to tumour progression [8]. The lack of these complex biochemical interplays in a 2D environment hence harbours a different microenvironmental pressure, causing cells cultured in 2D to adopt a different transcriptomic landscape [9,10] that eventually differs from that of derived tumours [11,12].

The pitfalls of 2D cultures have led to the rise in popularity of three-dimensional (3D) cultures for several reasons. For one, it is a relatively inexpensive tool that can serve as a bridge between highly restrictive 2D cultures and in vivo xenograft animal models, which have high cost, high maintenance, and also come bundled with increased ethics conundrums. 3D models have been postulated to possess the ability and genomic landscape to better mimic GBM tumour phenotypes, allowing for more robust drug evaluations [13]. In our recent scoping review, we reported on 45 genes (Table 1) that showed different expressions in 3D cultures when compared to 2D cultures, which are also investigated in the current study [14].

With the advent of technological advancements in the field of genomics, genetic data banks have continued to skyrocket in availability and size, with comprehensive data deposited in databases such as The Cancer Genome Atlas (TCGA), Gene Expression Omnibus (GEO), Chinese Glioma Genome Atlas (CGGA), and Genotype-Tissue Expression (GTEx), facilitating the evaluation of novel clinical hypotheses. While there are ample empirical data and investigations on clinical GBMs resulting from these databases, there have not been many analyses performed on the huge amount of transcriptomic data between preclinical GBM models and clinical GBM samples.

In this study, we analysed some of the huge genetic datasets that are available in databases to derive clinically relevant information regarding the transcriptomic landscape observed in 3D GBM cultures. We compared these with the transcriptomic data obtained from brain samples of GBM patients and normal/healthy subjects. We also combined the key findings from our previous analysis of 2D versus 3D cultures (Table 1) with several bioinformatic analyses, thus discovering the synonymous upregulation of genes found in 3D cultures that carries significant clinical significance in GBM patients. Hence, this paper provides evidence to support 3D GBM cultures as suitable preclinical models to more closely emulate GBMs in a clinical setting. Some definitive answers to the debate about the use of 2D and 3D cultures are also discussed.

## 2. Materials and Methods

### 2.1. RNA-Seq Expression Data

RNA-Seq expression data from GBM patients and healthy brain samples together with the clinical information from their respective datasets were downloaded from GEO (https://www.ncbi.nlm.nih.gov/geo/, accessed on 4 May 2022). Raw RNA-Seq expression counts for GSE147352 were provided and thus directly obtained from GEO [15]. For GSE145645 [16] and GSE165595 [17], raw RNA-Seq fastq files were downloaded using SRA Explorer in the absence of any provided raw RNA-Seq counts. To obtain raw RNA-Seq counts from the aforementioned expression datasets, STAR [18] was leveraged to align the RNA transcripts to Ensembl’s reference human genome (GRCh38 primary assembly). Quantifications were produced with the help of RSEM [19] using ENCODE3′s STAR-RSEM pipeline parameters. Paired-end alignments were performed for both datasets, with exact codes and all parameters used for the alignments being provided.

For validation, level 3 gene expression profiles of the TCGA GBM patient cohort and healthy brain samples were obtained from the UCSC Xena data portal (https://xenabrowser.net/datapages/, accessed on 16 May 2022) [20]. Specifically, Illumina Hiseq2000 RNA-Seq log2-transformed HTSeq counts were obtained. The clinical data of the GBM patients from the TCGA database, such as gender, age, *IDH1^R132^* mutation status, *MGMT* methylation status, sample type, survival, and outcome, were obtained from both UCSC Xena [20] and cBioPortal (https://www.cbioportal.org, accessed on 16 May 2022) [21,22]. Utilisation of the Toil pipeline allowed for a unified processing workflow between the TCGA and GTEx datasets, with STAR being used to generate alignments and quantifications obtained using RSEM [23]. The recomputation of the raw RNA-Seq data from the TCGA and GTEx datasets by the UCSC Xena project made the two datasets compatible, allowing for direct expression analyses. Following the acquisition of RNA-Seq data from the TCGA/GTEx cohort, raw RNA-Seq counts of healthy brain samples and GBM patients (Illumina Hiseq; mRNAseq_325 and mRNAseq_693) and the corresponding clinical information were also obtained from CGGA (http://www.cgga.org.cn/download.jsp, accessed on 27 November 2022) [24,25,26,27]. The CGGA database kindly provided RNA-Seq data for two batches (mRNAseq_325 and mRNAseq_693), and data from grade IV GBM patients from both batches were combined and analysed to further eliminate any batch effects. Table 2 summarises the information of the number of GBM patients and normal brain samples in each RNA-Seq cohort.

### 2.2. Identification of Differentially Expressed Genes

Differential expression analysis was performed in RStudio v4.1.1 using the ‘DESeq2’ [28] and ‘EnhancedVolcano’ packages [29]. |Log2 fold change (LFC)| > 1 and Benjamini–Hochberg adjusted *p*-values (padj) < 0.05 were set as the cut-offs for screening differentially expressed genes. All R scripts used for the differential expression analysis and subsequent analyses are provided (https://github.com/thegellerbing/2D_3D_Analysis-, accessed on 22 January 2023).

### 2.3. Construction of Gene Interaction Network

The interaction network was generated using STRING (Search Tool for the Retrieval of INteracting Genes/Proteins) v11 database (https://string-db.org/, accessed on 30 November 2022) [30]. The database predicts interactions between differentially expressed genes based on their physical binding and regulatory interactions. It analyses the network edges of evidence, confidence, and molecular actions between the genes. The minimum required interaction score was set at the highest confidence at 0.900.

### 2.4. Over-Representation Analysis

Over-representation analysis of the gene sets was performed using the R package ‘clusterprofiler’ [31]. Protein-coding genes that were evaluated in the differential expression analysis were used as the background. Gene annotations of biological processes that had a *p*-value less than 0.01 and a q-value adjusted using the Benjamini–Hochberg method of less than 0.05 were studied.

### 2.5. Survival Analysis

Kaplan–Meier survival analysis was performed on the TCGA cohort obtained from UCSC Xena. The analysis was performed in RStudio v4.1.1 using the ‘survival’ [32] and ‘survminer’ [33] packages. Patients from the TCGA cohort were dichotomised into high- and low-expression groups based on the median transcript per million (TPM) value. Patients in the CGGA cohort were stratified based on the fragments per kilobase of exon per million mapped fragments (FPKM) value. The overall survival of patients between the high- and low-gene-expression groups was compared using the log-rank test. A *p*-value of < 0.05 denoted a statistically significant result.

## 3. Results

### 3.1. Commonly Regulated Genes in 3D Cultures Are Replicated in GBM Patients

As a training set, RNA-Seq data of GBM patients compared to normal brain tissues from three different GEO datasets were compared. Out of the 45 genes that we found in our scoping review [14], 32, 35, and 22 genes were differentially expressed, respectively. Table 3 shows the subset of differentially expressed genes in each GEO dataset based on the genes listed in Table 1. Crucially, prominent stem cell markers (*PROM1*, *SOX2*, *NES)*, EMT markers (*FN1*, *CD44*), angiogenesis and migration markers (*VEGFA*, *MMP2*, *MMP9*), and hypoxia markers (*HIF1A*) were all upregulated in GBM patients compared to normal human brain samples. Other prominent EMT markers, such as *CDH2*, *TWIST1,* and *SNAI1,* as well as prominent migration marker *MMP1* were all upregulated in two of the GEO datasets bar GSE165595. It has been proposed that these markers are the reason for the innate aggressiveness of GBM, an observation that is replicated in 3D cultures when compared to traditional 2D cultures (Table 1). Naturally, even 3D GBM cultures will not be able to outright replicate the transcriptomic landscape of in vivo GBM tumours, with genes such as *ABCA2*, *CYP1A1*, and *EPCAM* being downregulated in GBM patients whereas increased expression of said genes is observed in 3D cultures. Conversely, the expression of *CCND1*, *CDC20*, *ITGA3,* and *MYC* genes was upregulated in GBM patients compared to normal brain samples, but these genes were downregulated in 3D cultures. The full list of differentially expressed genes for GSE145645, GSE165595 and GSE147352 cohorts can be found in Tables S1–S3, respectively.

To validate the results we obtained from GEO, RNA-Seq data of GBM patients were subsequently obtained from TCGA while RNA-Seq data of normal human brain samples from the cortex and frontal cortex were obtained from the GTEx repository. In total, the gene expression profiles and clinical information of 166 GBM patients were obtained from TCGA. Healthy human brain samples were obtained from both TCGA (*n* = 5) and GTEx (*n* = 207). Further validation was obtained from another large-scale sequencing study spearheaded by Zhao et al. [24], which resulted in the compilation of the CGGA database. This allowed differential expression analysis to be performed on brain samples from 388 GBM patients and 20 healthy individuals. The clinical characteristics of the 166 GBM samples from TCGA and the 388 GBM samples from the CGGA are summarised in Table 4.

**Figure 1:**
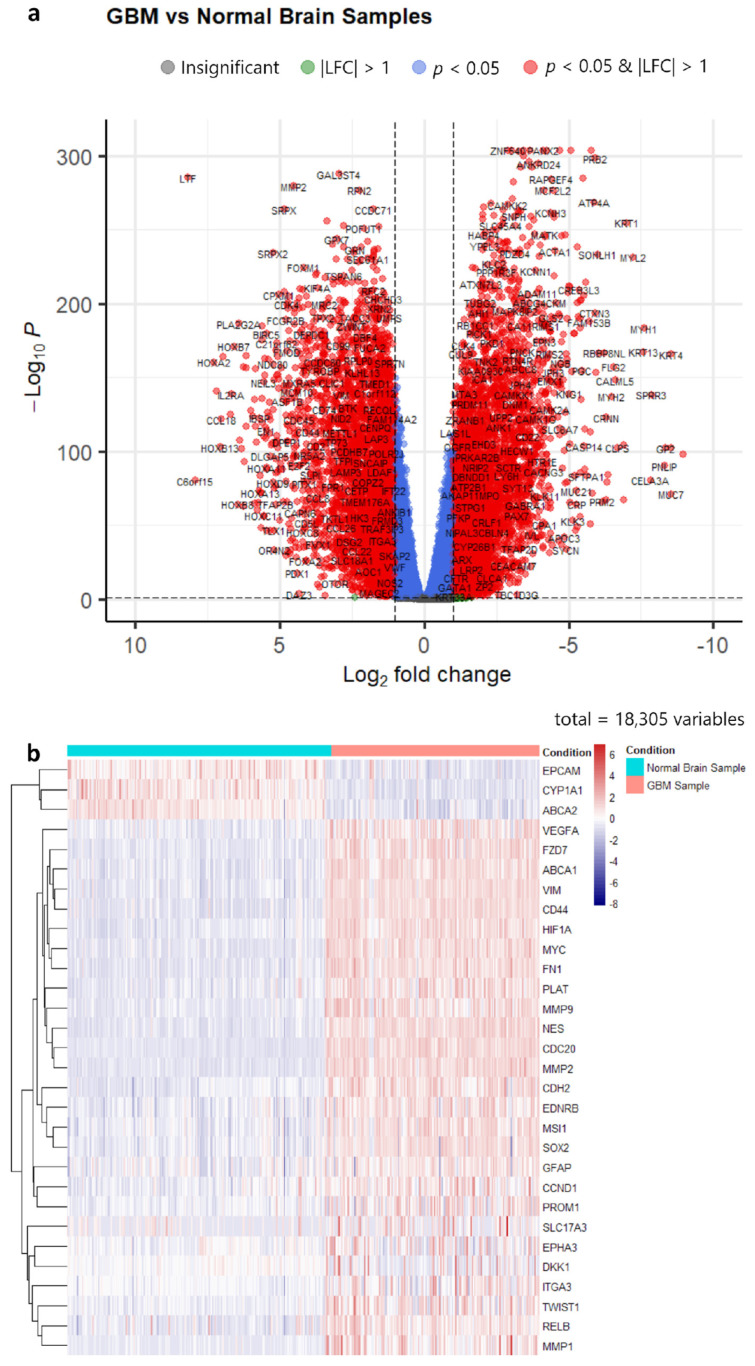
(**a**) Volcano plot of differentially expressed genes in the TCGA/GTEx cohort. (LFC = log2 fold change). (**b**) Heatmap of commonly regulated genes in 3D subset from Table 1 between GBM samples and normal brain tissues in the TCGA cohort.

**Figure 2:**
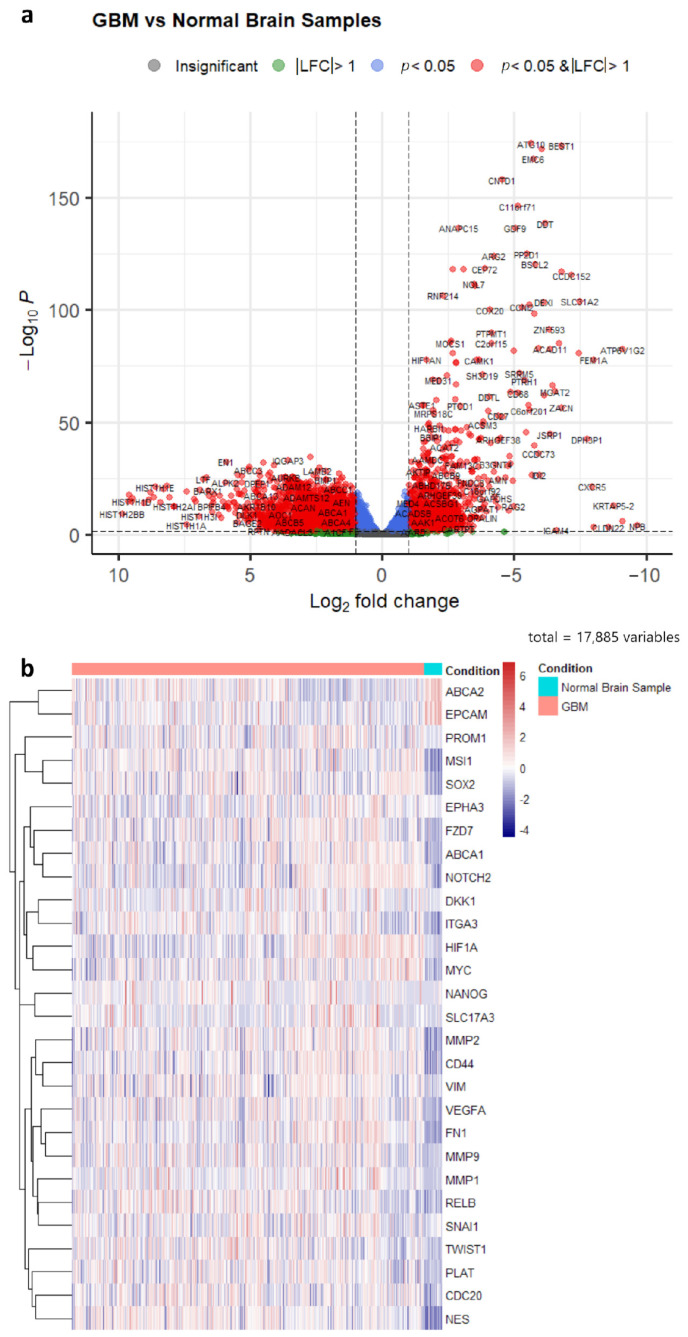
(**a**) Volcano plot of differentially expressed genes in the CGGA cohort (LFC = log2 fold change). (**b**) Heatmap of commonly regulated genes in 3D subset from Table 1 between GBM samples and normal brain tissues in the CGGA cohort.

**Figure 3:**
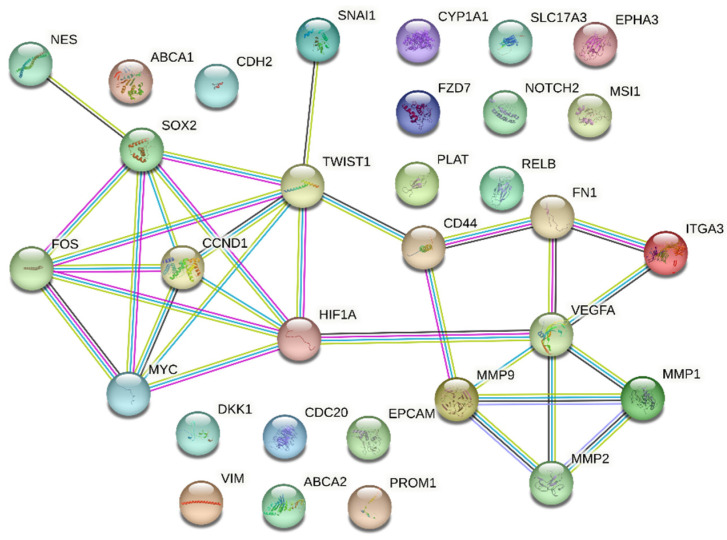
Interaction network of the subset of 31 genes from Table 1 that were differentially expressed between GBM samples and healthy brain samples in at least three or more cohorts. These represent genes with high-confidence interactions with each other.

From a total of 18,347 protein-coding genes, 6704 differentially expressed genes were identified in the TCGA and GTEx cohorts, with 3274 (18%) and 3457 (19%) genes being upregulated and downregulated in GBM patient samples, respectively (Table S4, Figure 1a). Furthermore, 30 of the genes listed in Table 1 met the log2 fold change threshold (padj < 0.05) (Table 5). Meanwhile, the differential expression analysis of the CGGA database saw a total of 3962 upregulated genes (22%) and 1852 downregulated genes (10%) out of 17,748 protein-coding genes (Table S5, Figure 2a), with 28 genes listed in Table 1 meeting the stated thresholds. Table 5 summarises the genes that were differentially expressed for the two different cohorts, while Figure 2b and Figure 3b show the different expression profiles of these differentially expressed genes in brain samples from GBM patients and healthy subjects.

A total of 14 genes were found to be upregulated across all five different cohorts, with most of the prominent stem cell markers, migration and angiogenic markers, EMT markers, and hypoxia markers being upregulated. Similarly, genes that heavily influence the mesenchymal state and migration of GBM (e.g., *TWIST1* and *MMP1*) were upregulated in both the TCGA/GTEx and CGGA cohorts (Table S6). In addition, several genes (*ABCA2*, *CYP1A1*, *EPCAM*, *CCND1*, *CDC20*, *ITGA3*, and *MYC*) that showed different expression profiles between 3D versus 2D cultures and GBM samples versus healthy brain samples in the GEO datasets were also replicated in the two larger cohorts.

A clear summary of the differentially expressed genes that were subset from genes listed in Table 1 are clearly shown in Table 6. With the exception of a couple of genes, the transcriptomic landscape of GBM patients is largely replicated by 3D cultures in vitro.

### 3.2. Differentially Expressed Genes in GBM Patients with Different Characteristics

Part of GBM’s aggressiveness stems from its heterogeneity. While GBM appears to be histopathologically similar across tumours, the molecular characteristics of GBM tumours differ from patient to patient. Several distinct molecular characteristics have dictated the response to therapy for most patients, with notable examples being the mutation status of *IDH1^R132^* and the methylation status of *MGMT* promoters. *IDH1^R132^* mutation status is now used as one of the characteristics to specify between the two grade 4 gliomas, namely GBMs with wild-type *IDH1^R132^* and *IDH1^R132^* mutant astrocytomas. GBMs with wild-type *IDH1^R132^* [34] and unmethylated *MGMT* promoters [35] are commonly associated with increased resistance against most therapeutic measures. Hence, we investigated whether there were any associations between the genes that were differentially expressed in 3D compared to 2D GBM cultures (Table 1) and the mentioned GBM molecular parameters to determine the effectiveness of 3D GBM cultures as a suitable treatment-resistant cell-based model.

For the GSE147352 cohort, only the *IDH1^R132^* mutation status of the GBM patients was available (n = 69), with 56 patients having wild-type *IDH1^R132^*s and 13 patients having mutant IDH1R132s. The differential expression analysis revealed that genes associated with stemness (*NES*, *PROM1*), EMT (*TWIST1*), angiogenesis and migration (*VEGFA*, *MMP9*), and genes involved in the Wnt signalling pathway (*DKK1*, *FZD7*) were upregulated (Table 7, |log2 fold change| >1, padj < 0.05). The GSE165595 cohort also provided information about the IDH1R132 mutation status (n = 17). A total of 15 GBM patients had wild-type *IDH1^R132^* while 2 GBM patients had mutant IDH1R132. However, differential expression analysis of said cohort yielded no significant results for any of the genes listed in Table 1.

Subsequently, we attempted to stratify GBM patients from the TCGA and CGGA cohorts based on *IDH1^R132^* mutation status (n = 144 and n = 378) and *MGMT* methylation status (n = 119 and n = 335). Any GBM samples that lacked the *IDH1^R132^* mutation and *MGMT* methylation status were excluded from the following analysis. We discovered nine genes listed in Table 1 that were upregulated in GBM patients with wild-type *IDH1^R132^* compared to *GBM* patients with mutant *IDH1^R132^* in the TCGA cohort. These genes are part of the Wnt signalling pathway (*FZD7*, *DKK1)* and are mainly involved in EMT (*CD44*, *SNAI1*) and migration/invasion (*MMP9*, *VEGFA)* (Table 8). The narrative followed through in the CGGA cohort, with notable genes such as *FN1*, *SNAI1*, *DKK1*, *FZD7*, *MMP1*, *MMP9*, and *VEGFA* having increased expression in wild-type *IDH* GBMs (Table 9).

Regarding GBM patients with unmethylated *MGMT* promoters, the expression of *TWIST1* and *MMP1* genes was found to be upregulated when compared to GBM patients with methylated *MGMT* promoters in the TCGA and CGGA cohorts, respectively. It is worth noting that GBM patients stratified in terms of their MGMT promoter methylation status yielded a smaller number of differentially expressed genes. More specifically, the TCGA and CGGA cohorts only reported 255 (0.014%) and 618 (0.035%) differentially expressed genes, respectively, between GBM patients with unmethylated and methylated *MGMT* status.

### 3.3. Gene Interactions among Differentially Expressed Genes

STRING was utilised to better understand the relationships between the identified differentially expressed genes found in GBM samples when compared to normal brain samples. The subset of 31 genes from Table 1 that were differentially expressed in at least three cohorts was used to construct the interaction network. Figure 3 shows the resulting interaction network between the genes. Notably, there are clear notable interactions between stemness markers (*NES*, *SOX2*, *FOS)*, hypoxia marker (*HIF1A),* EMT markers (*TWIST1*, *CD44*, *FN1)*, and migration/angiogenic markers (*VEGFA*, *MMP1*, *MMP2*, *MMP9)*. The mapped interaction chart seems to suggest a relationship between stemness, hypoxia, EMT, and the angiogenic process.

### 3.4. Functional Enrichment of the Differentially Expressed Genes

**Figure 4:**
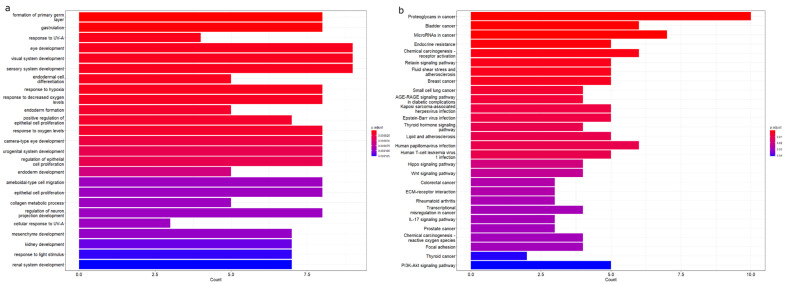
Overrepresentation analysis of enriched (**a**) GO biological processes and (**b**) KEGG pathways of the subset of 31 genes from Table 1 that were differentially expressed in at least three different cohorts.

To better understand the functions of all 31 genes from Table 1 that were differentially expressed in at least 3 different cohorts, overrepresentation analysis was performed with the 31 genes as a defined gene set. Figure 4a shows the top 30 GO biological processes. Some of the notable biological processes include response to hypoxia and mesenchyme development. The enriched KEGG pathways are displayed in Figure 4b. Table 10 further summarises the statistically significant GO biological process and KEGG pathway annotations involved in GBM development.

### 3.5. Correlation of Upregulated Genes with GBM Patients’ Overall Survival

**Figure 5:**
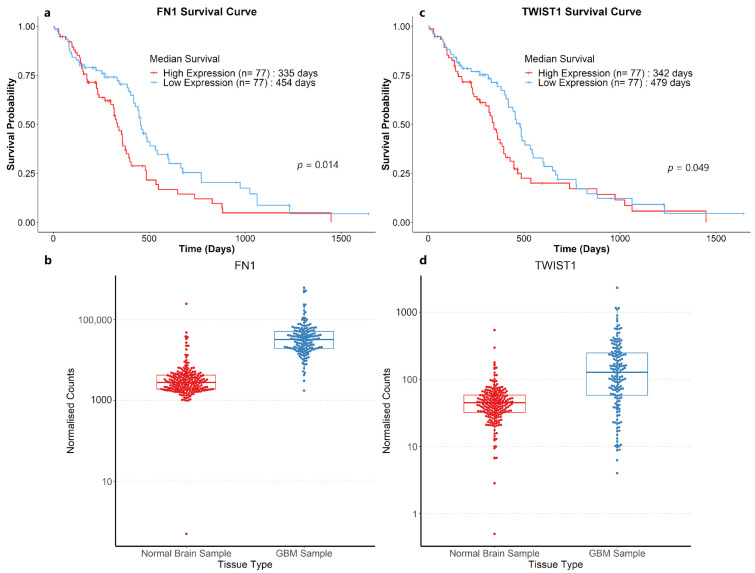
Survival curves of 163 GBM patients and count plots between normal brain samples and GBM samples for genes (**a**,**b**) *FN1* and (**c**,**d**) *TWIST1*. GBM patients were dichotomised into two groups for the log-rank tests based on the median TPM value of each respective gene.

To round out the study, Kaplan–Meier survival curves were plotted for genes that were upregulated in GBM samples based on the survival data provided by the TCGA and CGGA databases. Among the 27 genes that were upregulated in GBM patients compared to normal human brain samples within the TCGA cohort, *FN1* and *TWIST1* were significant predictors of poorer overall survival based on the log-rank test (*p* < 0.05, Figure 5). On the other hand, 27 genes were upregulated in the CGGA cohort and a run through the list of genes revealed that 19 genes were significant predictors of poorer overall survival. It would seem that several stemness markers, EMT markers, and angiogenic markers corresponded to lower survival probabilities among CGGA GBM patients (Table 11).

## 4. Discussion

A strong foundation is the key to success. In terms of cancer research, said foundation would refer to the models that are used in preclinical evaluations. In our previous scoping review, we suggested that the paradigms of the more simplistic and reductive nature of 2D GBM culture preclinical models should be shifted in favour of 3D GBM models [11]. The justification for that statement was based on our findings that genes crucial to GBM survival and growth, such as genes related to stemness, the EMT process, angiogenesis, migration, and hypoxia response, were commonly upregulated in 3D models, suggesting that 3D models better define the tumour’s innate aggressiveness. In this paper, we sought to justify our stance and to search for answers regarding the clinical relevance of preclinical 3D models by utilising the vast array of genomic databases available in the information vaults of GEO, TCGA, GTEx, and CGGA. Our differential expression analysis across GEO, TCGA/GTEx, and CGGA asserted our stance vis-à-vis the paradigm shift towards 3D models in preclinical evaluations. EMT-related genes (*CD44*, *TWIST1*, *CDH2*, *FN1*, *VIM*, *YAP1)*, angiogenesis/migration-related genes (*MMP1*, *MMP2*, *MMP9*, *VEGFA)*, hypoxia-related genes (*HIF1A*, *PLAT)*, stemness-related genes (*SOX2*, *PROM1*, *NES*, *FOS*), and genes involved in the Wnt signalling pathway (*DKK1*, *FZD7*) were all upregulated in GBM samples vs. normal brain samples in several cohorts, as seen in 3D cultures when compared to 2D cultures, which were verified in previous biological experiments [36,37,38,39,40].

These genes play a massive role in dictating GBM’s aggressive nature, with *NES* being a prominent marker of the primitive undifferentiated phenotypes of GBM stem cells. Able to self-renew and differentiate, GBM stem cells have often been cited to be the reason for GBM’s high relapse rate [41,42]. Driven by the Wnt signalling pathway, GBM stem cells often adopt mesenchymal phenotypes through the EMT process, increasing the cells’ migratory and invasive capabilities, which explains the increased expression of genes such as *MMP1*, *MMP2*, *MMP9,* and *VEGFA* that are involved in facilitating GBM invasion and the vascularisation process [43,44,45,46,47,48,49]. Looking into the GO biological processes, the involvement of *FZD7*, *DKK1*, *SOX2*, *CDH2*, *FN1*, *SNAI1*, *TWIST1*, *HIF1A*, *MMP1*, *MMP2*, and *MMP9* in the Wnt signalling pathway, mesenchyme development, and extracellular matrix disassembly corroborate their roles in the increased invasive capabilities of GBM cells.

Furthermore, Figure 3 also demonstrated the close relationship between the upregulated genes that were found in both GBM samples and 3D cultures. The interaction network showed the distinct relationship between the genes that facilitate the EMT process, such as transcription factor *TWIST1* [50] and cell surface glycoprotein *CD44* that governs GBM invasion [51], with *SOX2*. Additionally, the relationship between these genes extended to genes that code for essential collagenases and gelatinases, such as *MMP1*, *MMP2*, and *MMP9* [52], genes that facilitate vascularisation, such as *VEGFA* [44], and genes that code for extracellular glycoproteins that bind to membrane proteins, such as *FN1* [53]. This was followed by the presence of an interaction between *HIF1A, TWIST1,* and *VEGFA*, and reports have indicated that hypoxia is a potent inducer of the EMT process, a phenotype that is easily reproducible with the hypoxic core in 3D cultures [54]. These results cement the notion that 3D cultures are superior in mimicking GBM’s innate aggressiveness compared to 2D cultures, with the formation of a hypoxic core that closely imitates the quiescent nature of the core and aggressive mesenchymal phenotypes of GBMs.

Researchers have been trying to profile the molecular and mutation characteristics of GBMs for decades. This would allow for a more obvious harmonisation of patient cohorts, facilitating more accurate therapy response predictions in the effort to achieve personalised medicine in the future. The most basic of this molecular and phenotypic profiling can be found in the form of *IDH1^R132^* mutation and *MGMT* promoter methylation. As mentioned earlier, two GBM phenotypes play a role in predicting treatment response and overall survival [35,55]. Consequently, using the clinical data available in the databases, we set out to determine whether 3D GBM cultures can act as reductive models for GBMs with increased treatment resistance. In the process, we observed notable decreases in the expression of genes involved in the EMT process, Wnt signalling, and invasion, such as *CD44*, *TWIST1*, *MMP1*, *MMP9*, *VEGFA*, *FZD7*, *DKK1,* and *SNAI1* in *IDH1^R132^* mutant GBM in the GEO, CGGA, and TCGA/GTEx cohorts, whereas *TWIST1* and *MMP1* were upregulated in TCGA and CGGA GBM patients with unmethylated MGMT promoters, respectively. A pattern can be seen in the form of increased EMT and invasive behaviour being at the forefront of GBM patients with increased treatment resistance. The *IDH1^R132^* mutation is known to facilitate inhibition of the Wnt signalling pathway, resulting in a less aggressive phenotype, possibly perpetrated by reduced mesenchymal phenotypes within the tumour [56,57]. The transcription factors involved in the EMT process have been found to directly contribute to chemoresistance by modulating the expression of *MGMT* [58,59]. However, arguments have to be made regarding whether the methylation status of *MGMT* of GBM patients affects the transcriptomic landscape since the percentage of genes that are differentially expressed in both databases is minuscule to say the least. All the data seem to point to 3D GBM cultures being a suitable substitute for GBMs with wild-type *IDH1^R132^* in preclinical therapy evaluations. Considering most GBM cell lines contain wild-type *IDH1^R132^*, 3D models have the capability to further emphasise the resistant nature of said GBM subtypes.

All of these results drive home the need for wider adoption of 3D GBM models for preclinical evaluations. The upregulation of *CD44*, *FN1*, *MMP1*, *MMP9*, *SNAI1,* and *VEGFA*, especially within the wild-type *IDH1^R132^* GBM group, emphasises the suitability of 3D cultures as preclinical substitutes for treatment-resistant models by simulating the EMT process and increased migratory capacity. A move to 3D cultures in preclinical evaluations may well see increased efficiency in weeding out unsuitable anticancer agents, hopefully leading to better clinical evaluation translations for future candidates. Additionally, the PI3K/Akt signalling pathway plays a central role in cell division, migration, adhesion, differentiation, and apoptosis, with its notorious role in activating EGFRs. The close relationship between *VEGFA* and *FN1* (Figure 3) and its involvement in this highly centralising pathway not only signify the importance of inhibiting the said pathway, but it also speaks volume about the pertinence of 3D models for such studies. The enrichment of the Wnt signalling pathway as a result of increased *DKK1* and *FZD7* expression in GBM patients makes 3D models a prime candidate for preclinical Wnt inhibitory studies. Further evidence for the need for widespread adoption of 3D cultures in preclinical evaluation can be seen in the Kaplan–Meier survival graphs. *FN1* and *TWIST1*, which have been prominent in this discussion, presented a direct correlation between increased expression and decreased overall survival in the TCGA cohort, a fact that has been corroborated by other studies [60,61]. On the other hand, the CGGA cohort indicated a majority of the genes (*CD44*, *CDH2*, *FN1*, *FOS*, *MMP1*, *MMP2*, *MMP9*, *NES*, *SNAI1*, *VEGFA*, and *VIM*) that were repeatedly mentioned were similar predictors of poorer survival. The survival analysis of the CGGA cohort also suggested that 3D models can better simulate the increased difficulty of drug delivery to GBM tumours, where patients with increased *ABCA1* expression experience lower survival probabilities. Additionally, upregulation of the *ABCA1* gene responsible for the increased efflux of chemotherapeutic drugs in 3D cultures was also replicated in every clinical dataset analysed.

However, 3D cultures are not a perfect model by any means. As stated in our previous scoping review, 3D cultures might produce contradictory results for studies targeting the cell cycle [14]. Genes that regulate the cell cycle, such as *CDC20*, *MYC,* and *CCND1,* were indeed upregulated in GBM patients compared to normal human brain samples, but they were found to be downregulated in 3D cultures compared to 2D cultures. This is doubly important for *MYC* and *CCND1* considering their critical contribution to biological processes such as mesenchyme development, response to hypoxia, response to xenobiotic stimulus, and the Wnt signalling pathway, not to mention being involved in the PI3K-Akt signalling pathway. We postulate that an optimum size of around 200~350 μm for the spheroids in 3D cultures simulates the hypoxic core where cells are mostly quiescent, which might affect the cell cycle [62,63]. For said studies that involve the cell cycle, it might behove researchers to vigorously maintain the spheroid size below 200 μm (the diffusion limit for oxygen) in order to better emulate the intermediate layers [64] or procure patient-derived cells to better represent the genetic landscape. Our bioinformatic analysis of both databases clearly indicated increased expression of *ITGA3* in GBM samples and in GBM patients with wild-type *IDH1^R132^*, whereas our previous analysis found decreased expression in 3D cultures [14]. The contrast in results only serves to drive home the importance of the extracellular matrix dynamics in driving the genetic landscape of GBMs. To date, multiple support systems have been used for 3D GBM cultures, and it is imperative that the properties of the chosen scaffolds be properly considered for the most accurate representation of in vivo GBMs.

## 5. Conclusions

In this paper, we found evidence of genes commonly upregulated in 3D cultures being replicated in clinical samples. Genes that regulate the EMT process not only have significant prognostic value, but they also seem to be the driving force behind GBM archetypes with poorer treatment responses. In short, we conclude that 3D cultures are superior to 2D cultures, with transcriptomic landscapes that better mimic clinical GBMs, even those with increased treatment resistance (i.e., GBMs with wild-type *IDH^R132^*), by mimicking the highly aggressive EMT process that plagues most GBM patients. 

## Figures and Tables

**Table 1:** The common functions of regularly upregulated and downregulated genes in 3D GBM models compared to 2D GBM cultures found in our previous scoping review [11].

Genes	Function
Upregulated Genes	
*PROM1*	Stemness-Related
*NES*	
*SOX2*	
*TAZ*	
*POU5F1*	
*NANOG*	
*FOS*	
*MSI1*	
*CD44*	EMT-Related
*TWIST1*	
*SNAI1*	
*FN1*	
*VIM*	
*CDH2*	
*YAP1*	
*MMP1*	Angiogenesis/Migration
*MMP2*	
*MMP9*	
*VEGFA*	
*EPHA3*	
*ABCG2*	Drug Efflux
*ABCB1*	
*ABCA1*	
*ABCA2*	
*ABCC7*	
*SLC17A3*	
*GFAP*	ECM-Related
*ITGA6*	
*EPCAM*	
*HIF1A*	Hypoxia
*PLAT*	
*DKK1*	Wnt Signalling
*FZD7*	
*RELB*	Regulation of Gene Expression
*MAML1*	
*IKBKB*	NFκB Signalling
*CDKN1B*	Cell Cycle
*EDNRB*	Cell Division
*CYP1A1*	Drug Response
*NOTCH2*	Notch Signalling
Downregulated Genes	
*CDH1*	EMT-Related
*ITGA3*	ECM-Related
*CCND1*	Cell Cycle-Related
*CDC20*	
*MYC*	

**Table 2:** Number of GBM patients and healthy brain samples in each dataset used.

Dataset	GBM Patients	Healthy Brain Samples
GSE145645	32	3
GSE147352	85	15
GSE165595	17	17
CGGA	388	20
TCGA/GTEX	166	212

**Table 3:** Differentially expressed genes in each GEO dataset based on the genes listed in Table 1.

GSE145645	GSE147352	GSE165595
Upregulated Genes		
*ABCA1*	*ABCA1*	*ABCA1*
*CCND1*	*CCND1*	*CCND1*
*CD44*	*CD44*	*CD44*
*CDC20*	*CDC20*	*CDC20*
*CDH2*	*CDH1*	*FN1*
*EDNRB*	*CDH2*	*FOS*
*EPHA3*	*DKK1*	*FZD7*
*FN1*	*EDNRB*	*HIF1A*
*FOS*	*FN1*	*MMP2*
*FZD7*	*FOS*	*MMP9*
*HIF1A*	*FZD7*	*MSI1*
*ITGA3*	*GFAP*	*MYC*
*MAML1*	*HIF1A*	*NES*
*MMP1*	*IKBKB*	*PLAT*
*MMP2*	*ITGA3*	*PROM1*
*MMP9*	*ITGA6*	*RELB*
*MSI1*	*MMP1*	*SOX2*
*MYC*	*MMP2*	*VEGFA*
*NES*	*MMP9*	*VIM*
*NOTCH2*	*MSI1*	
*PLAT*	*MYC*	
*PROM1*	*NES*	
*RELB*	*NOTCH2*	
*SNAI1*	*PLAT*	
*SOX2*	*PROM1*	
*TWIST1*	*RELB*	
*VEGFA*	*SLC17A3*	
*VIM*	*SNAI1*	
*YAP1*	*SOX2*	
	*TWIST1*	
	*VEGFA*	
	*VIM*	
	*YAP1*	
Downregulated Genes		
*ABCA2*	*CYP1A1*	*ABCA2*
*EPCAM*	*EPCAM*	*EPCAM*
*CYP1A1*		*CYP1A1*

**Table 4:** Clinical characteristics of the TCGA and CGGA GBM patient cohorts.

	Total Number (n = 166)	Total Number (n = 388)
**Sex** **Male** **Female** **Not Reported**	106 59 1	235 153 -
**Age** **>65** **<65** **Not Reported**	101 54 11	38 350 -
**IDH Status** **Wild-Type** **Mutant** **Not Reported**	136 8 22	288 90 10
**MGMT Methylation Status** **Methylated** **Unmethylated** **Not Reported**	54 65 47	172 163 53
**Sample Type** **Primary** **Recurrent**	153 13	255 133
**Event** **Living** **Deceased** **Not Reported**	48 106 12	53 322 13

**Table 5:** Subset of differentially expressed genes from the TCGA/GTEx and CGGA cohorts based on Table 1.

TCGA/GTEx	CGGA
Upregulated Genes	
***ABCA1***	*ABCA1*
***CCND1***	*CD44*
***CD44***	*CDC20*
***CDC20***	*DKK1*
***CDH2***	*EPHA3*
***DKK1***	*FN1*
***EDNRB***	*FZD7*
***EPHA3***	*HIF1A*
***FN1***	*ITGA3*
***FZD7***	*MMP1*
***GFAP***	*MMP2*
***HIF1A***	*MMP9*
***ITGA3***	*MSI1*
***MMP1***	*MYC*
***MMP2***	*NANOG*
***MMP9***	*NES*
***MSI1***	*NOTCH2*
***MYC***	*PLAT*
***NES***	*PROM1*
***PLAT***	*RELB*
***PROM1***	*SLC17A3*
***RELB***	*SNAI1*
***SLC17A3***	*SOX2*
***SOX2***	*TWIST1*
***TWIST1***	*VEGFA*
***VEGFA***	*VIM*
***VIM***	
Downregulated Genes	
***ABCA2***	*ABCA2*
***CYP1A1***	*EPCAM*
***EPCAM***	

**Table 6:** The subset of genes based on Table 1 that are differentially expressed in 3D cultures vs. 2D cultures and across all datasets used in the differential expression analysis.

Genes	3D vs. 2D	GSE145645	GSE165595	GSE147352	CGGA	TCGA/GTEx	Gene Function
*FOS*	**↑**	**↑**	**↑**	**↑**			Stemness-Related
*MSI1*	**↑**	**↑**	**↑**	**↑**	**↑**	**↑**	
*NANOG*	**↑**				**↑**		
*NES*	**↑**	**↑**	**↑**	**↑**	**↑**	**↑**	
*PROM1*	**↑**	**↑**	**↑**	**↑**	**↑**	**↑**	
*SOX2*	**↑**	**↑**	**↑**	**↑**	**↑**	**↑**	
*CD44*	**↑**	**↑**	**↑**	**↑**	**↑**	**↑**	EMT-Related
*CDH2*	**↑**	**↑**		**↑**		**↑**	
*FN1*	**↑**	**↑**	**↑**	**↑**	**↑**	**↑**	
*SNAI1*	**↑**	**↑**		**↑**	**↑**		
*TWIST1*	**↑**	**↑**		**↑**	**↑**	**↑**	
*VIM*	**↑**	**↑**	**↑**	**↑**	**↑**	**↑**	
*YAP1*	**↑**	**↑**		**↑**			
*EPHA3*	**↑**	**↑**			**↑**	**↑**	Angiogenesis/Migration
*MMP1*	**↑**	**↑**		**↑**	**↑**	**↑**	
*MMP2*	**↑**	**↑**	**↑**	**↑**	**↑**	**↑**	
*MMP9*	**↑**	**↑**	**↑**	**↑**	**↑**	**↑**	
*VEGFA*	**↑**	**↑**	**↑**	**↑**	**↑**	**↑**	
*ABCA1*	**↑**	**↑**	**↑**	**↑**	**↑**	**↑**	Drug Efflux
*ABCA2*	**↑**	**↓**	**↓**		**↓**	**↓**	
*SLC17A3*	**↑**			**↑**	**↑**	**↑**	
*GFAP*	**↑**			**↑**		**↑**	ECM-Related
*ITGA6*	**↑**			**↑**			
*EPCAM*	**↑**	**↓**	**↓**	**↓**	**↓**	**↓**	
*ITGA3*	**↓**	**↑**		**↑**	**↑**	**↑**	
*HIF1A*	**↑**	**↑**	**↑**	**↑**	**↑**	**↑**	Hypoxia
*PLAT*	**↑**	**↑**	**↑**	**↑**	**↑**	**↑**	
*DKK1*	**↑**			**↑**	**↑**	**↑**	Wnt Signaling
*FZD7*	**↑**	**↑**	**↑**	**↑**	**↑**	**↑**	
*MAML1*	**↑**	**↑**					Regulation of Gene Expression
*RELB*	**↑**	**↑**	**↑**	**↑**	**↑**	**↑**	
*IKBKB*	**↑**			**↑**			NF-κB Signalling
*CCND1*	**↓**	**↑**	**↑**	**↑**		**↑**	Cell Cycle-Related
*CDC20*	**↓**	**↑**	**↑**	**↑**	**↑**	**↑**	
*MYC*	**↓**	**↑**	**↑**	**↑**	**↑**	**↑**	
*EDNRB*	**↑**	**↑**		**↑**		**↑**	Cell Division
*NOTCH2*	**↑**	**↑**		**↑**	**↑**		Notch Signaling
*CYP1A1*	**↑**	**↓**	**↓**	**↓**		**↓**	Drug Response

**↑** indicates upregulation of the gene, **↓** indicates downregulation of the gene.

**Table 7:** Differentially expressed genes between wild-type *IDH1*^R132^ GBM vs. *IDH1^R132^* mutant astrocytoma in the GSE147352 cohort.

*IDH1^R132^* Wild-Type vs. *IDH1^R132^* Mutant	
*NES*	Upregulated
*PROM1*	Upregulated
*TWIST1*	Upregulated
*VEGFA*	Upregulated
*MMP1*	Upregulated
*MMP9*	Upregulated
*PLAT*	Upregulated
*DKK1*	Upregulated
*FZD7*	Upregulated
*EPHA3*	Upregulated
*ITGA3*	Upregulated

**Table 8:** Differentially expressed genes between GBM samples with different characteristics in the TCGA cohort.

*IDH1^R132^* Wild-Type vs. *IDH1^R132^* Mutant	
*CD44*	Upregulated
*MMP9*	Upregulated
*VEGFA*	Upregulated
*ITGA3*	Upregulated
*PLAT*	Upregulated
*FZD7*	Upregulated
*SNAI1*	Upregulated
*SLC17A3*	Upregulated
*DKK1*	Upregulated
*EDNRB*	Downregulated
**Unmethylated *MGMT* GBMs vs. Methylated *MGMT* GBMs**	
*TWIST1*	Upregulated

**Table 9:** Differentially expressed genes between GBM samples with different characteristics in the CGGA cohort.

*IDH1^R132^* Wild-Type vs. *IDH1^R132^* Mutant	
*DKK1*	Upregulated
*FN1*	Upregulated
*FZD7*	Upregulated
*ITGA3*	Upregulated
*MMP1*	Upregulated
*MMP9*	Upregulated
*SLC17A3*	Upregulated
*SNAI1*	Upregulated
*VEGFA*	Upregulated
*CCND1*	Downregulated
**Unmethylated *MGMT* GBMs vs. Methylated *MGMT* GBMs**	
*MMP1*	Upregulated

**Table 10:** GBM development-related GO biological process categories of the 31 genes that were differentially expressed in at least three different cohorts.

**Biological Processes**	***p*-Value**	**FDR**	Genes **
Response to Hypoxia (GO:0001666)	8.51 × 10^−6^	4.31 × 10^−6^	*FOS*, *TWIST1*, *MMP2*, *VEGFA*, *HIF1A*, *PLAT*, *MYC*, *CYP1A1*
Mesenchyme Development (GO:0060485)	9.71 × 10^−5^	9.71 × 10^−5^	*CDH2*, *FN1*, *SNAI1*, *TWIST1*, *EPHA3*, *HIF1A*, *MYC*
Negative Regulation of DNA Damage Response (GP:0043518)	2.25 × 10^−6^	1.54 × 10^−4^	*CD44*, *SNAI1*, *TWIST1*
Mesenchymal Cell Differentiation (GO:0048762)	1.54 × 10^−4^	2.92 × 10^−4^	*CDH2*, *FN1*, *SNAI1*, *TWIST1*, *EPHA3*, *HIF1A*
Mesenchymal Cell Migration (GO:0090497)	2.92 × 10^−4^	2.94 × 10^−4^	*CDH2*, *FN1*, *TWIST1*, *HIF1A*
Response to Xenobiotic Stimulus	2.94 × 10^−4^	2.35 × 10^−4^	*FOS*, *MMP2*, *ABCA2*, *ITGA3*, *CCND1*, *MYC*, *CYP1A1*
Positive Regulation of MAPK Cascade (GO:0043410)	4.44 × 10^−4^	4 × 10^−4^	*SOX2*, *CD44*, *CDH2*, *VEGFA*, *DKK1*, *FZD7*, *NOTCH2*
Stem Cell Development (GO:0048864)	7.52 × 10^−4^	7.52 × 10^−4^	*CDH2*, *FN1*, *TWIST1*, *HIF1A*
Wnt Signalling Pathway (GO:0016055)	2.75 × 10^−3^	1.46 × 10^−3^	*SOX2*, *CDH2*, *ITGA3*, *DKK1*, *FZD7*, *CCND1*
Positive Regulation of Epithelial Cell Migration (GO:0010634)	2.89 × 10^−3^	1.5 × 10^−3^	*MMP9*, *VEGFA*, *ITGA3*, *HIF1A*
Extracellular Matrix Disassembly (GO:0022617)	3.22 × 10^−3^	1.7 × 10^−3^	*MMP1*, *MMP2*, *MMP9*
Epithelial to Mesenchymal Transition (GO:0001837)	3.34 × 10^−3^	1.8 × 10^−3^	*SNAI1*, *TWIST1*, *EPHA3*, *HIF1A*
**KEGG Pathway**	***p*-Value**	**FDR**	Genes **
MicroRNAs in Cancer	6.58 × 10^−5^	4.4 × 10^−6^	*MMP9*, *CD44*, *VIM*, *MYC*, *VEGFA*, *CCND1*, *NOTCH2*
Wnt Signalling Pathway	3.04 × 10^−3^	0.023	*FZD7*, *MYC*, *DKK1*, *CCND1*
ECM-Receptor Interaction	3.98 × 10^−3^	0.027	*CD44*, *FN1*, *ITGA3*
Transcriptional Misregulation in Cancer	4.94 × 10^−3^	0.029	*MMP9*, *MYC*, *PLAT*, *PROM1*
PI3K-Akt Signalling Pathway	8.63 × 10^−3^	0.028	*FN1*, *MYC*, *VEGFA*, *ITGA3*, *CCND1*

** Genes were analysed via overrepresentation analysis using the R package ‘clusterprofiler’ and only categories with False Discovery Rate (FDR)-adjusted *p*-value of < 0.05 related to cancer development are reported.

**Table 11:** Univariate survival analysis of 374 CGGA GBM patients based on the upregulation of genes in GBM patients compared to normal human brain samples. Patients were dichotomised into two groups based on the median FPKM value.

Gene	Hazard Ratio	95% CI	*p*-Value
*ABCA1*	1.402785	1.13~1.75	0.002547
*CD44*	1.408099	1.13~1.76	0.002341
*CDC20*	1.26485	1.01~1.58	0.036394
*CDH2*	1.323025	1.06~1.65	0.012566
*FN1*	1.403916	1.13~1.75	0.002561
*FOS*	1.382786	1.11~1.72	0.003926
*ITGA3*	1.304676	1.05~1.63	0.01783
*MMP1*	1.249728	1~1.56	0.046397
*MMP2*	1.308285	1.05~1.63	0.016909
*MMP9*	1.249362	1~1.56	0.04784
*MSI1*	1.306559	1.05~1.63	0.017031
*MYC*	1.289035	1.04~1.61	0.023265
*NES*	1.337433	1.07~1.67	0.009639
*PLAT*	1.407802	1.13~1.76	0.002363
*RELB*	1.250162	1~1.56	0.046568
*SNAI1*	1.421935	1.14~1.77	0.001759
*VEGFA*	1.353984	1.09~1.69	0.006891
*VIM*	1.422233	1.14~1.77	0.00175
*YAP1*	1.248786	1~1.55	0.047023

## Data Availability

All alignment scripts and R scripts used in this paper can be accessed through the following link: https://github.com/thegellerbing/2D_3D_Analysis- (accessed on 22 January 2023).

## Supplementary Materials

The following supporting information can be downloaded at: https://www.mdpi.com/article/10.3390/biology12050648/s1, Table S1: Significant differentially expressed genes between GBM samples and healthy brain samples for the GSE145645 cohort; Table S2: Significant differentially expressed genes between GBM samples and healthy brain samples for the GSE165595 cohort; Table S3: Significant differentially expressed genes between GBM samples and healthy brain samples for the GSE147352 cohort; Table S4: Significant differentially expressed genes between GBM samples and healthy brain samples for TCGA/GTEx cohort; Table S5: Significant differentially expressed genes between GBM samples and healthy brain samples for the CGGA cohort. Table S6: Significant differentially expressed genes subset from Table 1 between GBM samples and healthy brain samples for every cohort.
