# Supplementary material for: Identification of Prominent Genes between 3D Glioblastoma Models and Clinical Samples via GEO/TCGA/CGGA Data Analysis
Source: Biology (Basel). 2023 Apr 25;12(5):648. doi: 10.3390/biology12050648 (PMC10215494; doi:10.3390/biology12050648)
Supplement: Supplementary file 1 [file biology-12-00648-s001.zip › Supp Table 6 - Significant differentially expressed genes subset from Table 1 between GBM samples and healthy brain samples for every cohort.pdf]

Differentially expressed genes subset from Table 1 between GBM samples and normal human brain samples for GSE147352 cohort

Genes	Base Mean	Log_2 Fold Change	Standard Error	P-Value	Adjusted P-Value
MSI1	365.427	1.600	0.184	2.98E-18	2.25952E-17
PROM1	600.309	2.227	0.390	1.11E-08	3.45907E-08
SOX2	4043.760	2.285	0.167	2.2E-42	1.46831E-40
FOS	5111.737	2.803	0.278	6.23E-24	8.14372E-23
NES	9004.037	4.274	0.218	3.33E-85	5.48616E-82
CDH2	4659.289	1.033	0.151	7.74E-12	3.24347E-11
YAP1	1487.710	1.111	0.238	3.01E-06	7.2978E-06
TWIST1	55.231	1.165	0.382	0.002302	0.004099778
SNAI1	28.874	1.255	0.388	0.001201	0.002212754
CDH1	160.248	1.348	0.428	0.001617	0.002935569
VIM	28611.616	3.404	0.258	8.17E-40	4.25242E-38
FN1	42415.464	3.970	0.335	2.06E-32	5.717E-31
CD44	10530.386	4.450	0.358	2.17E-35	7.68035E-34
VEGFA	12206.480	3.456	0.440	3.78E-15	2.17167E-14
MMP2	970.520	4.599	0.253	6.87E-74	5.40984E-71
MMP9	448.979	6.098	0.644	2.65E-21	2.67612E-20
MMP1	24.532	6.768	0.986	6.63E-12	2.79988E-11
SLC17A3	1.179	2.551	0.987	0.009706	0.015928398
ABCA1	10508.517	3.689	0.212	4.08E-68	1.8014E-65
EPCAM	22.837	-1.431	0.438	0.001103	0.002043346
ITGA6	2210.811	1.124	0.172	6.27E-11	2.40667E-10
ITGA3	1920.299	1.303	0.369	0.000421	0.000819338
GFAP	131414.20	1.875	0.260	5.44E-13	2.54251E-12
HIF1A	7956.446	1.967	0.202	1.77E-22	1.9833E-21
PLAT	1123.477	2.378	0.326	2.96E-13	1.41949E-12
DKK1	99.418	1.600	0.651	0.014054	0.022506316
FZD7	621.124	3.112	0.322	3.78E-22	4.10374E-21
RELB	148.546	2.205	0.267	1.32E-16	8.64453E-16
IKBKB	1155.791	1.010	0.099	3.08E-24	4.13214E-23
EDNRB	4603.684	2.164	0.294	1.78E-13	8.66994E-13
CYP1A1	3.302	-3.212	0.491	5.94E-11	2.28651E-10
NOTCH2	7356.405	1.327	0.123	4.83E-27	8.3496E-26
CCND1	1654.017	1.664	0.209	1.86E-15	1.09692E-14
MYC	625.901	3.127	0.246	7.16E-37	2.80661E-35
CDC20	225.090	5.159	0.342	1.54E-51	2.10923E-49

Differentially expressed genes subset from Table 1 between GBM samples and normal human brain samples for GSE165595 cohort

Genes	baseMean	log2FoldChange	lfcSE	stat	pvalue	padj
PROM1	495.8884	1.721612	0.40823	4.217264	2.47E-05	0.000154
VIM	16706.06	2.501187	0.296032	8.449042	2.94E-17	3.01E-14
CD44	2900.971	2.508063	0.413564	6.064515	1.32E-09	3.95E-08
MMP2	893.1754	2.76511	0.386592	7.152526	8.52E-13	1.03E-10
HIF1A	4409.994	1.265628	0.21691	5.834798	5.39E-09	1.26E-07
MMP9	248.8063	5.482611	0.678319	8.082647	6.34E-16	2.69E-13
PLAT	1205.915	2.056544	0.4165	4.937678	7.91E-07	8.36E-06
RELB	84.6391	1.11862	0.295069	3.791053	0.00015	0.000716
CCND1	1820.973	2.052512	0.430785	4.764588	1.89E-06	1.77E-05
VEGFA	4298.785	3.102866	0.462671	6.706422	1.99E-11	1.31E-09
FN1	16070.67	2.807638	0.36962	7.596021	3.05E-14	6.65E-12
CDC20	145.8782	2.078644	0.496306	4.188227	2.81E-05	0.000171
NES	4841.741	2.978093	0.34616	8.603233	7.75E-18	9.65E-15
MSI1	325.707	1.092504	0.32271	3.385404	0.000711	0.002675
MYC	407.5988	1.967223	0.354746	5.545447	2.93E-08	5.2E-07
FZD7	311.3016	1.835188	0.406265	4.517219	6.27E-06	4.78E-05
ABCA1	5614.806	1.969887	0.279276	7.053556	1.74E-12	1.85E-10
FOS	6202.133	1.40004	0.497351	2.814993	0.004878	0.013952
SOX2	3842.545	1.346616	0.233654	5.763284	8.25E-09	1.8E-07
ABCA2	18377.9	-1.18912	0.453429	-2.62251	0.008729	0.022723
EPCAM	43.69772	-1.75367	0.390058	-4.49592	6.93E-06	5.2E-05
CYP1A1	5.630444	-2.12141	0.523197	-4.0547	5.02E-05	0.00028

Differentially expressed genes subset from Table 1 between GBM samples and normal human brain samples for GSE145645 cohort

hgnc_symbol	baseMean	log2FoldChange	lfcSE	stat	pvalue	padj
ITGA3	2737.164	1.68959	0.729726	2.315377	0.020592	0.067251111
PROM1	431.1779	1.568249	0.761862	2.058442	0.039548	0.108192165
VIM	69334.12	3.535413	0.520915	6.786927	1.15E-11	1.2562E-08
CD44	9735.676	4.254388	0.690442	6.161828	7.19E-10	2.15076E-07
EPHA3	376.2735	1.68872	0.927668	1.820392	0.068699	0.160422611
MMP2	2516.42	4.056793	0.645982	6.280041	3.38E-10	1.1847E-07
HIF1A	4017.58	1.763467	0.596652	2.955602	0.003121	0.017448585
MMP9	1972.685	8.940527	1.264796	7.068752	1.56E-12	2.75036E-09
PLAT	2283.135	2.167816	0.650802	3.33099	0.000865	0.006991869
RELB	395.5863	1.672682	0.533379	3.136013	0.001713	0.011383608
CCND1	2753.365	2.065364	0.652308	3.166239	0.001544	0.010553701
VEGFA	21584.22	5.422657	0.817221	6.635481	3.23E-11	2.21695E-08
FN1	46110.03	4.873751	0.82051	5.939903	2.85E-09	6.17287E-07
CDC20	599.9744	1.972903	0.657291	3.001569	0.002686	0.01570126
TWIST1	239.3278	2.538446	1.019515	2.489857	0.012779	0.048048771
SNAI1	92.91465	1.577746	0.755185	2.089218	0.036688	0.10248256
NES	19459.93	3.451291	0.509847	6.769272	1.29E-11	1.33073E-08
NOTCH2	3384.489	1.303963	0.441057	2.95645	0.003112	0.017418435
MSI1	598.8694	1.051277	0.53514	1.964488	0.049473	0.1267464
EDNRB	3607.917	1.182401	0.784207	1.507766	0.131614	0.254174329
MYC	950.3779	3.3114	0.623305	5.312648	1.08E-07	9.98494E-06
YAP1	1350.055	1.318062	0.515012	2.559282	0.010489	0.041909551
FZD7	1149.693	3.64153	0.787033	4.626912	3.71E-06	0.000132642
MAML1	1142.333	1.070408	0.345394	3.099094	0.001941	0.012468453
ABCA1	3062.636	2.735342	0.591278	4.62615	3.73E-06	0.000132642
FOS	10234.22	1.583615	0.579256	2.733878	0.006259	0.028847436
CDH2	3410.738	1.242829	0.617298	2.013336	0.044079	0.116726292
SOX2	6341.05	1.34547	0.667194	2.016609	0.043736	0.116229828
MMP1	76.3496	8.570636	1.924702	4.452969	8.47E-06	0.000241033
ABCA2	11737.5	-1.73386	0.60329	-2.87401	0.004053	0.021058726
EPCAM	19.47253	-1.66687	0.686518	-2.42801	0.015182	0.054375165
CYP1A1	3.884225	-1.20248	1.02439	-1.17385	0.240456	0.387641445

Differentially expressed genes subset from Table 1 between GBM samples and normal human brain samples from TCGA and GTEx cohorts

Genes	Base Mean	Log_2 Fold Change	Standard Error	P-Value	Adjusted P-Value
MSI1	684.659	1.235	0.074	4.2E-62	1.54E-61
SOX2	5369.511	1.655	0.086	1.8E-82	9.07E-82
PROM1	480.546	1.817	0.119	3.2E-52	1.02E-51
NES	10302.656	3.485	0.115	5E-200	3.3E-198
CDH2	3329.602	1.375	0.060	7E-115	6.2E-114
TWIST1	118.313	2.005	0.125	9.5E-58	3.28E-57
VIM	52543.434	2.875	0.114	2E-140	2.6E-139
FN1	23945.952	3.500	0.127	6E-168	1.8E-166
CD44	8624.218	4.024	0.176	4E-115	4E-114
EPHA3	348.505	1.237	0.127	2.7E-22	5.16E-22
VEGFA	6999.937	2.533	0.131	2E-83	1.06E-82
MMP2	2122.176	4.516	0.126	3E-283	2.2E-280
MMP9	686.053	4.721	0.214	3E-108	2.7E-107
MMP1	27.644	5.000	0.249	2.3E-89	1.31E-88
ABCA2	14333.387	-1.806	0.112	1E-58	3.56E-58
SLC17A3	1.663	1.964	0.274	8.2E-13	1.32E-12
ABCA1	1793.344	2.546	0.096	1E-155	2.5E-154
GFAP	273024.03	1.227	0.121	4.3E-24	8.7E-24
ITGA3	2087.488	1.437	0.107	7.1E-41	1.88E-40
EPCAM	54.123	-1.676	0.136	4.4E-35	1.05E-34
HIF1A	4779.627	1.925	0.079	2E-132	2.6E-131
PLAT	1679.987	2.060	0.106	5.5E-84	2.88E-83
DKK1	149.307	2.477	0.194	3.5E-37	8.74E-37
FZD7	735.004	2.979	0.113	7E-154	1.5E-152
RELB	300.990	1.285	0.097	6E-40	1.58E-39
CYP1A1	22.694	-3.105	0.152	4.4E-93	2.71E-92
EDNRB	3342.969	1.736	0.108	4.5E-58	1.55E-57
CCND1	1678.414	1.183	0.100	3.9E-32	8.86E-32
MYC	700.588	2.548	0.120	3E-99	1.83E-98
CDC20	444.122	4.126	0.160	3E-146	5.7E-145

Differentially expressed genes subset from Table 1 between GBM samples and normal human brain samples from the CGGA cohort

hgnc	baseMean	log2FoldChange	lfcSE	stat	pvalue	padj
ABCA1	3296.29	1.867467	0.27362	6.825042	8.79E-12	1.08E-10
CD44	6592.768	1.874523	0.306911	6.107706	1.01E-09	8.47E-09
CDC20	570.7743	2.124772	0.299574	7.092641	1.32E-12	1.84E-11
DKK1	225.4809	2.780371	0.569256	4.884216	1.04E-06	4.7E-06
EPHA3	330.1231	1.469742	0.417349	3.52161	0.000429	0.00112
FN1	27391.97	3.579212	0.351472	10.18348	2.35E-24	1.49E-22
FZD7	920.5372	1.865717	0.319239	5.844266	5.09E-09	3.73E-08
HIF1A	3483.941	1.065234	0.222888	4.779236	1.76E-06	7.6E-06
ITGA3	2473.208	2.668451	0.30978	8.614011	7.05E-18	2.12E-16
MMP1	35.08582	5.038056	0.678733	7.422737	1.15E-13	1.88E-12
MMP2	2243.063	2.3504	0.259257	9.065896	1.24E-19	4.64E-18
MMP9	1358.535	3.910336	0.495688	7.8887	3.05E-15	6.37E-14
MSI1	974.6912	1.7736	0.194531	9.11731	7.7E-20	2.97E-18
MYC	993.5962	1.741867	0.261557	6.659602	2.75E-11	3.07E-10
NANOG	2.188561	2.264307	0.776694	2.915314	0.003553	0.007469
NES	23478.98	2.902763	0.285911	10.1527	3.22E-24	2E-22
NOTCH2	2855.467	1.073746	0.207164	5.18308	2.18E-07	1.15E-06
PLAT	1802.927	2.062796	0.284849	7.241708	4.43E-13	6.62E-12
PROM1	369.4774	1.323225	0.336927	3.927333	8.59E-05	0.000261
RELB	469.9257	1.66434	0.237365	7.011723	2.35E-12	3.15E-11
SLC17A3	3.724706	2.565394	0.771156	3.326686	0.000879	0.002123
SNAI1	75.15742	1.065058	0.30836	3.453945	0.000552	0.001408
SOX2	8210.367	1.330622	0.190496	6.985026	2.85E-12	3.74E-11
TWIST1	245.1913	2.449195	0.355292	6.893463	5.45E-12	6.92E-11
VEGFA	14767.58	3.745874	0.39945	9.377571	6.75E-21	2.97E-19
VIM	24890.14	1.213019	0.279665	4.337403	1.44E-05	5.14E-05
ABCA2	11511.3	-1.34129	0.289264	-4.63691	3.54E-06	1.43E-05
EPCAM	39.27876	-1.79746	0.361417	-4.97336	6.58E-07	3.12E-06
